# Acute Serum Amyloid A induces cell migration cytoskeletal rearrangement and Notch signalling in rheumatoid arthritis

**DOI:** 10.1186/ar3611

**Published:** 2012-02-09

**Authors:** Mary Connolly, Peadar Rooney, Wei Gao, Douglas Veale, Ursula Fearon

**Affiliations:** 1 Translational Research Group, Dublin Academic Medical Centre, St. Vincent's University Hospital, Dublin, Ireland

## Background

Acute Serum Amyloid A (A-SAA) is an acute phase protein strongly expressed in rheumatoid arthritis (RA) synovial tissue (ST) critically involved in regulating cell migration and angiogenesis. These processes are dependent on downstream interactions between extracellular matrix and cytoskeletal components. Additionally the Notch signalling pathway has been show to regulate endothelial cell (EC) morphogenesis and is critically involved in vessel formation, branching and morphogenesis. The aim of this study was to examine if A-SAA-induced angiogenesis, cell migration and invasion are mediated by the NOTCH signalling pathways.

## Materials and methods

Immunohistology was used to examine Notch1, DLL-4 and HRT-1 in RA synovial tissue (RAST). αvβ3 and β1-integrins,filamentous actin (F-actin) and focal adhesion expression in RAST and rheumatoid arthritis synovial fibroblast cells (RASFC) was assessed by immunofluorescence. NOTCH1 IC, its ligands DLL-4, JAGGED 1 and downstream signaling components HRT1, HRT2 were quantified by Real-time PCR. NOTCH1 IC protein was assessed by western blot. A-SAA-induced angiogenesis cell migration and invasion were assessed by Matrigel tube formation, scratch and invasion assay. A-SAA modulation of filamentous actin (F-actin) and focal adhesions (vinculin) was examined by dual immunofluorescence. Finally, A-SAA-induced angiogenesis, invasion, altered cell shape and migration were performed in the presence or absence of siRNA against NOTCH 1.

## Results

Notch1 and its ligands DLL-4 and HRT-1 were expressed in RAST both in the lining layer and perivascular regions. Additionally αvβ3, β1-integrin and F-actin predominantly localised to vascular endothelium and lining cells in RAST, compared with osteoarthritis and normal control synovial tissue. A-SAA significantly upregulated levels of Notch1 mRNA and protein in ECs. Differential effects were observed on Notch ligands HRT-1 and Jagged 1 mRNA in response to A-SAA stimulation. In contrast, A-SAA inhibited DLL-4 mRNA (p < 0.05), consistent with a negative feedback loop controlling interactions between NOTCH1 IC and DLL-4 in the regulation of EC tip vs. stalk cells development. A-SAA induced disassembly of endothelial cell F-actin cytoskeleton and loss of focal adhesions as demonstrated by a reduction in vinculin staining. Finally, A-SAA-induced angiogenesis, cell migration and invasion were inhibited in the presence of NOTCH 1 siRNA (p < 0.05).

## Conclusion

A-SAA induces the NOTCH signalling pathway and cytoskeletal rearrangement which allows temporal and spatial reorganization of cells during cell migratory events and EC morphology. Together these results suggest a critical role for A-SAA in driving cell shape, migration and invasion in the inflamed joint.

